# Method for assessing the content of molybdenum enzymes in the internal organs of fish

**DOI:** 10.1016/j.mex.2024.102576

**Published:** 2024-01-19

**Authors:** Mereke Satkanov, Diana Tazhibay, Bibigul Zhumabekova, Gulmira Assylbekova, Nurzhan Abdukarimov, Zhadyrassyn Nurbekova, Maral Kulatayeva, Karlygash Aubakirova, Zerekbai Alikulov

**Affiliations:** a L.N. Gumilyov Eurasian National University, Department of Biotechnology and Microbiology, Astana, 010000, Kazakhstan; b Immanuel Kant Baltic Federal University, Higher School of Living Systems, Kaliningrad, 236041, Russia; c Pavlodar Pedagogical University, Higher School of Natural Science, Pavlodar, 140002, Kazakhstan; d Nazarbayev University, School of Medicine, Astana, 010000, Kazakhstan

**Keywords:** Method for fish biochemistry, Molybdenum cofactor, *Neurospora crassa* nit-1, Fish molybdenum enzymes, Fish liver enzymes, Fish intestines enzymes, Assays molybdenum enzymes in the internal organs of fish

## Abstract

Molybdenum enzymes (Mo-enzymes) contain a molybdenum cofactor (MoCo) in the active site. These enzymes are potentially interesting for studying the survival mechanism of fish under hypoxic water conditions. This is because Mo-enzymes can synthesize nitric oxide from nitrates and nitrites, which are present in high concentrations under hypoxic water conditions. However, there is currently no method for assessing the Mo-enzymes content in the fish internal organs. Methods capable of determining Mo-enzymes content in the fish are of major importance. For this purpose, a method for quantitative determination of MoCo from plant tissues was modified. We demonstrated the Mo-enzyme content assessment by isolated MoCo from the fish's internal organs and the *Neurospora crassa* nit-1 extract containing inactive NADPH nitrate reductase. The Mo enzyme content was calculated using a calibration curve in nM of nitrites as a product of restored NADPH reductase activity after complementation with MoCo. Here we present a robust laboratory method which can be used to assess the content of Mo-enzymes in the internal organs of fish.

• Mo-enzymes play a crucial role in detoxifying toxic compounds. Therefore, it is important to develop a method to accurately determine the amount of Mo-enzymes present. Notably, the method demonstrated the efficiency and accuracy as detected high content of Mo-enzymes in the liver and intestines (*P* < 0.0001). The obtained data on the distribution of Mo-enzymes in the internal organs of this species correspond to that of other vertebrates. Here, we present a rapid, sensitive, accurate and accessible method.
• The developed method is simple and easy to use. Importantly, the protocol does not require complex manipulations, and the equipment used is available in most laboratories. The article provides step-by-step instructions for reproducing the method.

## Graphical abstract

Graphical abstract

**Figure:**
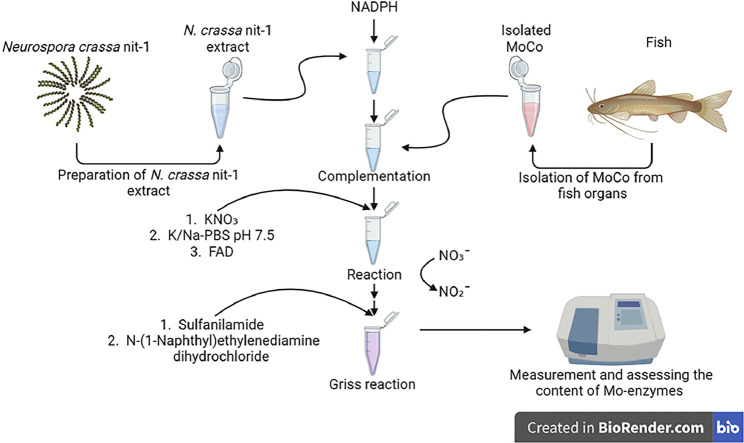

Specifications table

**Table:** 

Subject area:	Biochemistry, genetics and molecular biology
More specific subject area:	Fish molybdenum enzymes
Name of your method:	Assays molybdenum enzymes in the internal organs of fish
Name and reference of original method:	Mendel, R.R., Müller, A.J. Reconstitution of NADH-nitrate reductase *in vitro* from nitrate reductase-deficient Nicotiana tabacum mutants. Molec. Gen. Genet. 161, 77–80 (1978). https://doi.org/10.1007/BF00266617
Resource availability:	Reagents and Equipment are listed in the Method details

## Method details

Recent studies have highlighted the significant role of molybdenum enzymes (Mo-enzymes) in the fish body. According to Aubakirova et al., Mo-enzymes can convert nitrates and nitrites into nitric oxide (NO) [1]. This mechanism has been suggested to be involved in hypoxia, which is supported by previous studies indicating that NO-synthase-independent production of NO by Mo-enzymes is activated precisely during hypoxia [2]. Furthermore, Hansen and Jensen demonstrated a decrease in nitrates and nitrites concentration during hypoxia and the preservation of NO levels in fish, despite the main enzyme involved in the nitrogen oxide synthesis (NO synthase) not being able to fully function at insufficient levels of oxygen [3,4]. However, to our knowledge, there is still limited information on the distribution of Mo-enzymes, including molybdenum cofactor (MoCo), in the fish's internal organs. The absence of a method for assessing the Mo-enzymes content in the fish internal organs may have contributed to the lack of research in this area. Therefore, this study aimed to develop a method for assessing the content of Mo-enzymes in the internal organs of fish by using MoCo and an extract of the fungus *Neurospora crassa* (*N. crassa*) nit-1. To develop the method, we significantly modified the protocol which previously used to determine MoCo in plants [5].

According to Wang et al., *N. crassa* is a heterothallic filamentous fungus that is commonly used in genetic and biochemical research [6]. A mutant strain of *N. crassa* nit-1 has a mutation in the nit-1 gene associated with the synthesis of MoCo, rendering it unable to synthesize this cofactor [7]. Lee et al. previously postulated that MoCo acts as a link between protein subunits of Mo-enzymes and serves as an electron carrier [8]. The method used in this study involves complementing isolated MoCo from various fish organs with an extract of *N. crassa* nit-1 containing NADPH nitrate reductase. The enzymatic activity is then assessed in Units (nM product/min). This method represents the first biochemical method for assessing Mo-enzymes content in the fish body. The effectiveness of this method was verified through a series of analyzes using aquaponics-grown African sharptooth catfish (*Clarias gariepinus*). This method can be used to study the distribution of Mo-enzymes in the fish body under various stress and normal conditions.

## African sharptooth catfish organs preparation

Organs of the African sharptooth catfish were provided by the Laboratory of Aquaponics and the Study of Hydrobionts at the L.N. Gumilyov Eurasian National University. African sharptooth catfish were reared under aquaponic conditions for two months. Fish of both sexes were used in this study. The average pH of the water was 7.5 ± 0.5, total ammonia nitrogen - 0.048 mg/L, nitrite - 0.92 mg/L, and nitrate - 20 mg/L. The fish were mortified by using the exsanguination method [9]. After dissection and harvesting, the organs of the African sharptooth catfish were frozen in liquid nitrogen and stored at -80 °C.

## *Neurospora crassa* nit-1 cultivation

*N. crassa nit-1* was obtained from the collection of microorganisms at the University of Braunschweig. Cultivation was performed according to the method described by Mendel et al. with some modifications [5,10]. Fries medium No. 3 was used for mutant fungus cultivation [11]. During the preparation of the culture medium, the NH₄ tartrate was replaced by potassium sodium tartrate to make ammonium nitrate as the sole nitrogen source. In a solid medium, cultivation was carried out for 48 h at 32 °C. Cultivation in a liquid medium was carried out in an orbital shaker at 150 rpm, 32 °C, with constant heating for 72 h.

## *N. crassa* nit-1 extract preparation

Vegetative hyphae of *N. crassa* nit-1 were filtered through a ceramic filter using a vacuum pump. The *N. crassa* nit-1 filtrated hyphae were ground in a ceramic mortar with liquid nitrogen. This was followed by homogenisation buffer A: K/Na-phosphate buffer 0.05 M pH 7.0 with 5 mM ethylenediaminetetraacetic acid (EDTA), 1 % NaCl, and 1 mM phenylmethanesulfonyl fluoride (PMSF) [5,10,12]. The resulting homogeneous mass was exposed to ultrasound for 60 s at the maximum power setting of a Hielscher ultrasonic device UP200S. Efficient cooling with crushed ice was utilized during the treatment. The extract of nit-1 was obtained by centrifugation of the homogenate at 20,000 rpm for 20 min at 4 °C. The obtained supernatant was used for further experiments.

## МоСо isolation from fish internal organs

To isolate MoCo from internal organs, the first cell-free extract was obtained. Buffer B was prepared for this purpose. Buffer B contains 5 mM reduced glutathione, 25 mM Na₂MoO₄ and has a different pH (7.6), 0.05 M K/Na-phosphate buffer, 5 mM EDTA, and 1 % NaCl [5,12]. Organs were measured and ground in a ceramic mortar using liquid nitrogen. Buffer B (freshly prepared) was added at a ratio of 1:3 w/v. The resulting homogenate was treated for 60 s at maximum power on a Hielscher ultrasonic device UP200S with efficient cooling with crushed ice. Thus, a cell-free extract was obtained. This extract was centrifuged at 20,000 rpm for 10 min at +4 °C.

The second step is the heat treatment of the MoCo source. The heat treatment described by Mendel included a thorough evacuation step, nitrogen rinsing, plugging, incubation for 90 s at 70 °C in a water bath, and immediate cooling on ice [12]. In this method, buffer B contained 5 mM reduced glutathione to release MoCo. Therefore, the heat treatment steps were reduced, and the temperature requirements were changed. According to Alikulov and Schiemann, anaerobic and aerobic incubation in the presence of glutathione gave comparable results [13]. Thus, the heat treatment for the isolation of MoCo from the cell-free extract was performed under aerobic conditions in a water bath at 80 °C for 3 min and cooled on ice. The precipitated protein was removed by centrifugation at 5000 rpm for 5 min at +4 °C. The resulting supernatant was used as a source of MoCo for the assessment of Mo-enzymes content.

## Assessing the Mo-enzymes content

The content of Mo-enzymes was assessed by restoring the activity of NADPH nitrate reductase in *N. crassa* nit-1 extracts using MoCo isolated from internal organs. For this, apoenzyme was the complementation with the cofactor [5]. To the 100 μl of the nit-1 extract, 10 μl of 10 mM NADPH was added and then incubated for 5 min at room temperature. Then 50 µl of MoCo source was added and incubated for 15 min at room temperature.

In the second stage, the reaction of reduction of nitrate (NO₃¯) to nitrite (NO₂¯) was carried out [13]. For this purpose, 50 µl of KNO₃ 0.1 M (source of NO₃¯) was added, and 190 µl of 0.05 M K/Na-phosphate buffer at pH 7.5 was also added. The reaction was started by adding 100 μl of 1 mM FAD. The reaction mixture was then incubated at room temperature for 20 min. The reaction was stopped by boiling for 6 min at 100 °C in a water bath. Denatured proteins were removed by centrifugation for 5 min at 5000 rpm at +4 °C. Following, we determined the content of NO₂¯.

The content of NO₂¯ was determined spectrophotometrically using the Griess reaction. For this, 500 µl of 1 % sulphanilamide (SA) which dissolved in 20 % HCl was added to 500 µl reaction mixture, followed by 500 µl of 0.12 % N-l-naphthylethylenediamine-diHCl (NEDD) dissolved in distilled water [1]. The reaction proceeds with the formation of a purple azo dye. The color intensity is measured spectrophotometrically at 548 nm. The quantitative determination of the content of NO₂¯ was carried out by constructing the calibration curve. The calculation was made according to the calibration curve.

This method is based on the spontaneous insertion of a cofactor into NADPH nitrate reductase from *N. crassa* nit-1 *in vitro* [14]. One unit of MoCo restores the NADPH nitrate reductase activity in nit-1 extract to produce 1 nM nitrite per minute [5,10,12,13]. The Mo-enzymes content was estimated (one unit of activity) by the formation of 1 nM nitrite in 1 min.

## Experiment scheme

During the study, one blank, two negative controls, and one positive control were used for the determination of the content of NO₂¯. The positive control was used to validate the experimental procedure. The control solution contained buffer A, 10 μl of distilled water, and 50 μl of buffer B. The first negative control (C+) contained 100 μl of buffer A, 10 μl of 10 mM NADPH and 50 μl of MoCo source. The second negative control contained 100 µl of the nit-1 extract, 10 µl of 10 mM NADPH, and 50 µl of buffer B (C++). These controls were used to confirm that the reaction was not spontaneous. The results of the first negative control were subtracted from the values. Commercial bovine XO (Grade I, ammonium sulfate suspension, ≥0.4 units/mg protein) from SIGMA was used as a positive control. For complementation, XO diluted in buffer B (10 μl of XO in 990 μl of buffer B) was used instead of the MoCo source.

## Data analysis and calculation

The calculation to assess the Mo-enzymes content was performed according to the formula taking into account the absorbance of the control solution at 548 nm. The Mo-enzymes content was expressed in Units. The calculation was performed in several steps. In the first step, the blank value was subtracted from the sample value of all samples used (Eq. 1).

(1) $$X=ODsample-\;\bar{OD}control$$

where X is the absorbance value, which is used to calculate the nitrite concentration; ODsample *-* sample absorbance at 548 nm; $\bar{OD}control$ *-* average value of the blank absorbance at 548 nm;

In the second step, the concentration of nitrites (*ConcNO₂¯*) in nM was calculated from the X values obtained using the formula of the calibration curve. In the third step, the content of Mo-enzymes was calculated in Units (Eq. 1) by dividing the concentration of nitrite in nM (product) by the reaction time (in minutes).

(2) $$Mo-enzymes=\;\frac{ConcNO_{2}^{-}}{t}$$

where, Mo-enzymes - activity of NADPH nitrate reductase in Units (product concentration/minute); *ConcNO₂¯-* concentration of nitrites in nM; t - Reaction time from the addition of FAD to incubation at 100 °C in a water bath.

The statistical analysis was performed using the GraphPad 8 program with ANOVA (ordinary) and Tukey's multiple comparatives test. Prior to ANOVA, the normality of the data was checked using the Kolmogorov-Smirnov test. The P-value of less than 0.05 was considered statistically significant (*P* < 0.05) [15].

## Description of the experimental procedure

**Fig. 1:**
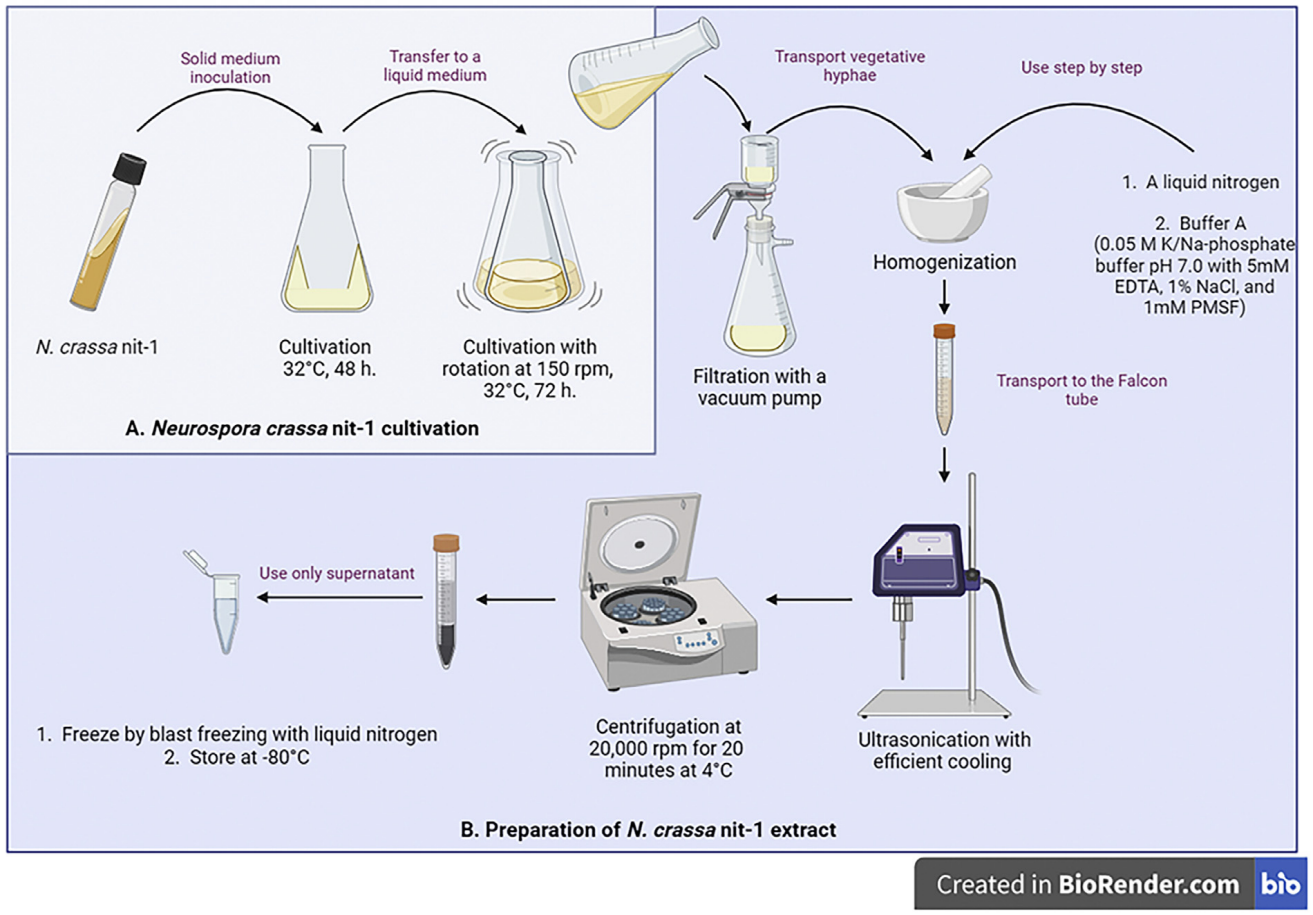
*N. crassa* nit-1 cultivation and extract preparation. Created with BioRender.com. All stages are described in the text. EDTA - Ethylenediaminetetraacetic acid; PMSF – phenylmethanesulfonylfluoride.

**Fig. 2:**
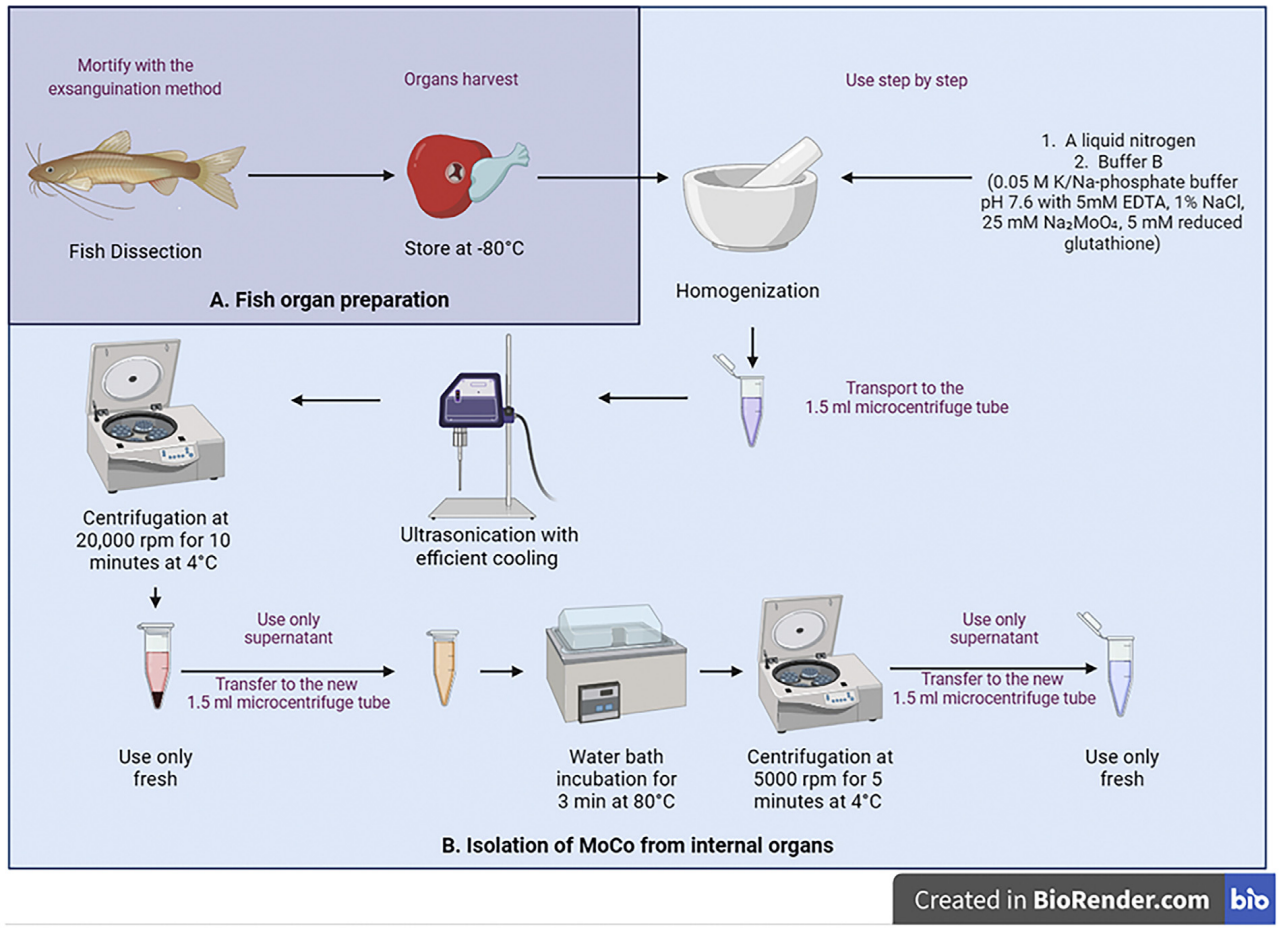
Fish organ preparation and MoCo isolation. Created with BioRender.com. All stages are described in the text. EDTA - Ethylenediaminetetraacetic acid.

**Fig. 3:**
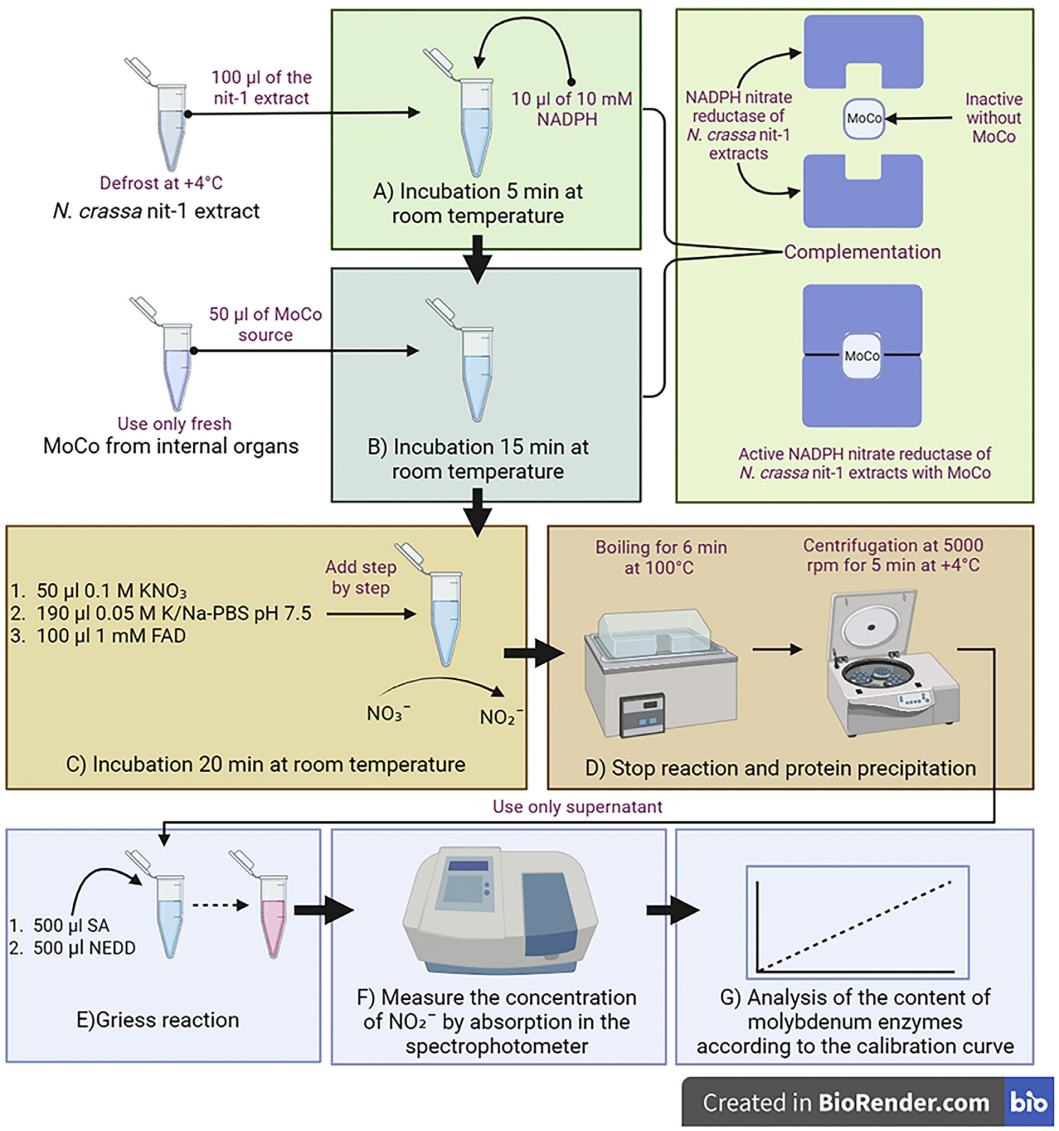
Assessment of molybdenum enzyme content in fish internal organs. Created with BioRender.com. All stages are described in the text. NADPH - Nicotinamide adenine dinucleotide phosphate; FAD - Flavin adenine dinucleotide; K/Na-PBS – Potassium sodium phosphate-buffered saline; SA – sulphanilamide; NEDD - N-l-naphthylethylenediamine-diHCl; NO₃¯ - nitrate; NO₂¯ - nitrite.

The study modified the method for the determination of the MoCo content in plants for determining the content of Mo-enzymes in the internal organs of fish [5]. The procedure consists of two stages: preparation of *N. crassa* nit-1 extract (Fig. 1) and MoCo isolation from internal organs (Fig. 2), followed by a reaction to assess the content of Mo-enzymes (Fig. 3).

The preparation phase was divided into four stages. The first stage was to demonstrate the cultivation of *N. crassa* nit-1 (Fig. 1A). It is recommended that 3 % agar is added to the medium to improve adhesion to the walls of the Erlenmeyer flask. 1–1.5 % agar did not produce uniform solidification. It is also advisable to exclude any source of nitrogen other than NH₄ tartrate, as a visual slowing of the growth of the fungus on a solid medium has been observed with other sources of nitrogen. Fries medium No. 3 can be changed to Vogel's medium N as the nutrient medium for *N. crassa* cultivation [11]. All procedures must be carried out under aseptic conditions. The cultivation time of the mutant fungus can vary. In our experiment, we obtained the best yield of the mutant fungus when cultivated for 48 h in the solid nutrient medium and 72 h when cultivated in the liquid nutrient medium.

In the second stage (Fig. 1B), an *N. crassa* nit-1 extract was prepared, which contains inactive NADPH nitrate reductase [13]. To filter vegetative hyphae after cultivation in the liquid medium, a ceramic filter should be used along with a vacuum pump. Homogenization should be done using a ceramic mortar and liquid nitrogen. Once the vegetative hyphae are ground to a powder, the buffer should be added. To prevent partial degradation and loss of activity of NADPH nitrate reductase, PMSF, a protease inhibitor, was added to buffer B [16]. Homogenization on the sonicator should be carried out at a temperature in the range of +4–8 °C to prevent protein denaturation [17]. If the sample becomes foamy or hot during sonication, it can lead to protein denaturation [17]. In our case, to obtain the clear supernatant, we precipitate the sediment at maximum centrifuge speed. The centrifugation mode can be changed.

The third stage (Fig. 2A) involves mortifying the fish, dissecting it, and preparing its organs. It is suggested to avoid using a mortifying method that causes severe hypoxia. This is because Mo-enzymes may be involved in hypoxia and could produce unreliable results [1]. The organs can be used immediately after collection for MoCo isolation or frozen at -80 °C. The fourth stage (Fig. 2B) involves isolating MoCo from internal organs. Homogenization is carried out using a buffer containing 5 mM reduced glutathione and 25 mM Na₂MoO₄ to facilitate the isolation process. The original method suggested creating anaerobic conditions, but adding 5 mM reduced glutathione allowed the reaction to proceed under aerobic conditions [5]. Both anaerobic and aerobic incubation with reduced glutathione yield comparable results [13]. Once the denatured proteins have precipitated, the MoCo source should be used promptly within 15 min to obtain a more accurate result.

After preparing of *N. crassa* nit-1 extract and MoCo from internal organs, a reaction was performed to assess the Mo-enzymes content (Fig. 3). The *N. crassa n*it-1 extract, stored at -80 °C, should be thawed on ice or at +4 °C in the refrigerator. The MoCo source should be prepared freshly. The experiment began by incubating *N. crassa* nit-1 extract, which contains NADPH nitrate reductase, with NADPH at room temperature for 5 min (Fig. 3A). NADPH acts as an electron donor for nitrate reductase [18]. The MoCo source was added to the mixture and incubated at room temperature for 15 min (Fig. 3B). Mixing *N. crassa* nit-1 extract and MoCo source in the presence of NADPH complements the apoenzyme with the cofactor [5,10,12,13]. Complementation with bioavailable MoCo spontaneously converts inactive NADPH nitrate reductase into its active form [19,20].

The next step involved adding the substrate (NO₃¯), K/Na-phosphate buffer to maintain a constant pH, and FAD (Fig. 3C). The reaction began after the FAD addition and proceeded at room temperature for 20 min. The reaction resulted in the formation of NO₂¯ from NO₃¯ [5]. To stop the reaction, it was boiled in a water bath at 100 °C for 6 min (Fig. 3D). It is worth noting that NADPH strongly inhibits the Griess reaction, which was used to determine the content of NO₂¯ [21]. Therefore, it is necessary to stop the reaction by boiling as NADPH degrades at high temperatures [22]. Skipping this step may result in irrelevant outcomes. Following incubation in the boiling water bath, the denatured proteins were precipitated through centrifugation (Fig. 3D). Subsequently, the Griess reaction was performed using SA and NEDD at room temperature (Fig. 3E). The color intensity was measured using a spectrophotometer at 548 nm after 10 min (Fig. 3F). The Mo-enzymes content was assessed following the methods described (Fig. 3G).

## The method validation

**Fig. 4:**
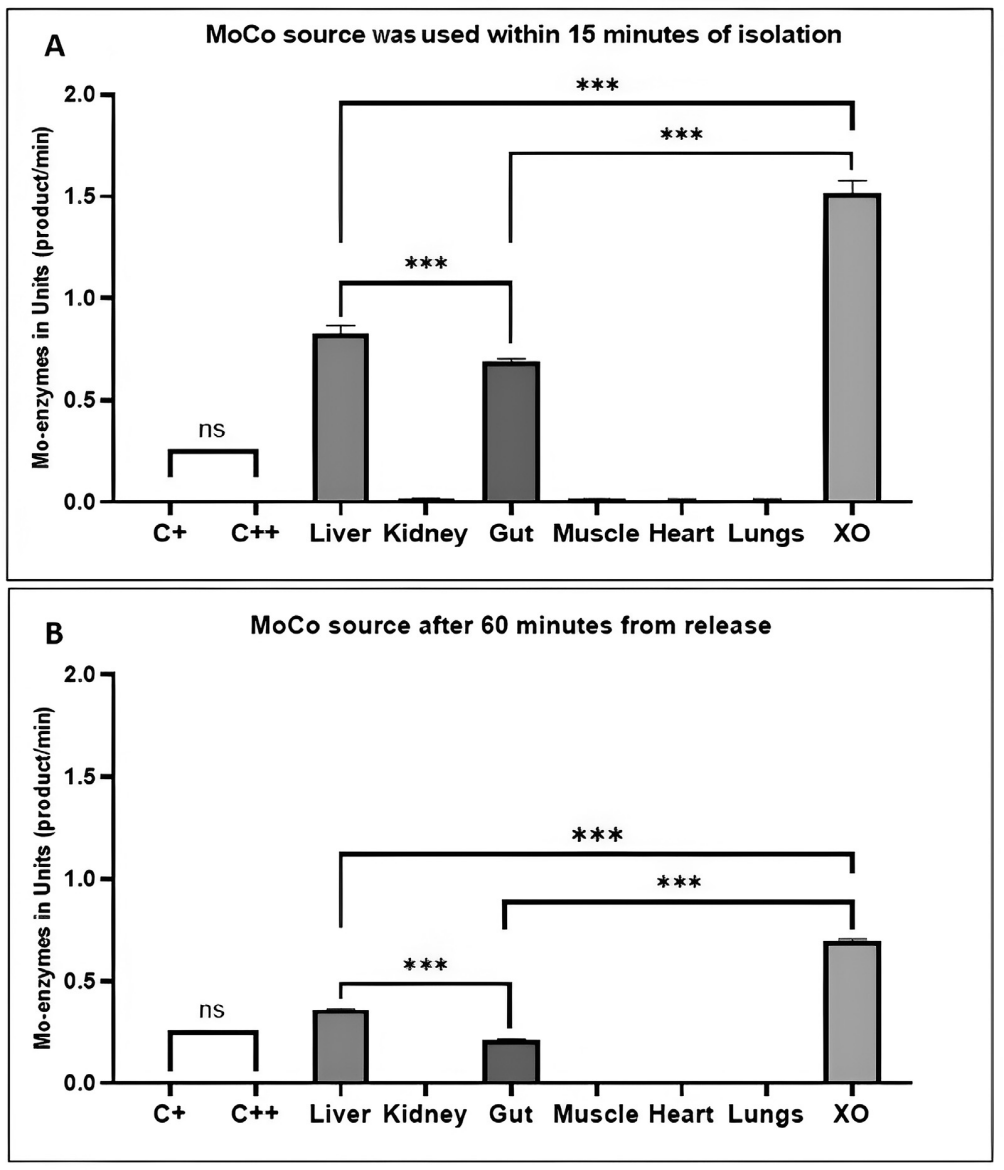
The Mo-enzymes content (Units) in the various fish internal organs. C+ - first negative control; C++ - second negative control; XO – positive control; *** - *P* < 0.0001; ns - non-significant.

The method's effectiveness was confirmed through a series of analyzes on African sharptooth catfish organs (Fig. 4). The data in Fig. 4A was obtained under the specified conditions, using a MoCo source within 15 min of isolation (Fig. 4А). The study revealed that the liver and intestines of the fish contained the highest concentration of Mo-enzymes (*P* < 0.0001, Fig. 4А). The liver contained 20.24 % more Mo-enzymes than the intestines (*P* < 0.0001). However, the kidneys, heart, and lungs had only 1.66 % of the Mo-enzymes content found in the liver (*P* < 0.0001).

We highly recommend using freshly prepared MoCo sources. Long-term storage, defined as more than 60 min, can partially degrade the cofactor. The results of this degradation are shown in Fig. 4B (*P* < 0.0001). It has been determined that after 15 min, the reaction activity disappears. This may be due to MoCo degradation, as mentioned earlier it is not a stable compound [23]. The reaction activity was significantly reduced not only in specimens isolated from African sharptooth catfish organs but also in commercial XO. MoCo from XO that was isolated 60 min prior showed only 53.99 % reactivity compared with MoCo from XO that was used within 15 min of isolation. Our experimental procedure demonstrated that when MoCo was used after 60 min in organs with low Mo-enzymes content, the intensity of the reaction was the same as in negative controls.

It was determined that the African sharptooth catfish has the highest content of Mo-enzymes in the liver (Fig. 3). Like other vertebrates, the fish liver is the main organ for detoxifying xenobiotics [24]. It is also the primary metabolic organ for energy storage and mobilization, performing several anabolic and catabolic functions [24]. The XO family of Mo-enzymes exhibits specificity towards a wide range of substrates, indicating their involvement in the metabolism of various xenobiotics [25,[26], [27], [28]]. Early studies by Aubakirova et al. have supported the high content of liver Mo-enzymes in *Silurus glanis* liver [1]. As hepatocytes are in direct contact with the bloodstream, they can absorb blood-borne chemicals, including nitrates and nitrites [24]. According to Aubakirova et al., the Mo-enzymes in the fish liver might participate in the nitrogen compounds removal from blood [1].

Several researchers have mentioned the high content of Mo-enzyme - XO in the intestines of humans and rats [[29], [30], [31]]. The fish intestine is evolutionarily adapted to cope with large amounts of water containing dissolved NO₃¯ and NO₂¯ [32,33]. A high content of Mo-enzymes in the intestine may be associated with the metabolism of these nitrogen compounds. Furthermore, the gut acts as a barrier against infectious pathogens and harmful substances [34]. Mo-enzymes are present in large amounts in fish intestines and can generate reactive oxygen species, which may protect against pathogens [[34], [35], [36]]. According to Juan et al.*,* all major Mo-enzymes found in animals can synthesize reactive oxygen species [34]. Additionally, recent research has shown that Arabidopsis thaliana aldehyde oxidase 3 (AAO3) can detoxify toxic aldehyde compounds and protect plants from early senescence, supporting the role of Mo-enzymes in detoxification [36]. Minor amounts of the Mo-enzyme are detected in all organs of fish since these enzymes are expressed by all types of eukaryotic cells [19,[37], [38], [39]]. It is suggested that these enzymes belong to the XO family.

## Conclusion

To address the challenge of evaluating Mo-enzyme levels in animals, we have developed an effective method using fish as a model. Our paper presents a comprehensive approach to assessing Mo-enzyme content, including principles, components, and step-by-step stages. We have demonstrated the effectiveness of our method by applying it to the internal organs of the fish. Our findings indicate that Mo-enzyme levels vary across different organs of the fish. The method used revealed that the liver and intestines of the fish contained the highest amount of Mo-enzymes, while the kidneys, heart, and lungs showed a lower level of Mo-enzymes. Our developed method has significant potential for rapid detection and assessment of Mo-enzymes content in fish organs. It is simple, cost-effective, and well-suited for environments with limited resources where advanced laboratory facilities may be unavailable. Furthermore, the simplicity and effectiveness of this method will make a significant contribution to the research field related to Mo-enzyme content in animals and address important questions. In summary, this method is a valuable and essential tool for the rapid and effective detection and assessment of Mo-enzymes content.

## Ethics statements

The ethics governing the use and conduct of experiments on animals were strictly observed, and the experimental protocol was approved by the L.N. Gumilyov Eurasian National University (Committee on Natural Science Research Ethics, approval number 1, July 2023).

## CRediT authorship contribution statement

**Mereke Satkanov:** Conceptualization, Methodology, Investigation, Validation, Data curation, Writing – original draft, Visualization, Supervision. **Diana Tazhibay:** Methodology, Investigation, Validation. **Bibigul Zhumabekova:** Conceptualization, Validation, Writing – review & editing. **Gulmira Assylbekova:** Conceptualization, Validation, Writing – review & editing. **Nurzhan Abdukarimov:** Writing – original draft, Visualization, Writing – review & editing. **Zhadyrassyn Nurbekova:** Writing – original draft, Writing – review & editing. **Maral Kulatayeva:** Project administration, Data curation. **Karlygash Aubakirova:** Project administration, Data curation. **Zerekbai Alikulov:** Conceptualization, Methodology, Investigation, Supervision.

## Declaration of competing interest

The authors declare that they have no known competing financial interests or personal relationships that could have appeared to influence the work reported in this paper.

## Data Availability

All data supporting the findings of this study are available within the paper.
